# Colonization success of a tree‐killing bark beetle: Geographic variation and mismatch with host preference

**DOI:** 10.1002/ece3.10274

**Published:** 2023-07-05

**Authors:** Etsuro Takagi

**Affiliations:** ^1^ Department of Tourism Science Tokyo Metropolitan University Tokyo Japan

**Keywords:** *Abies*, biogeographic boundary, colonization success, *Polygraphus proximus*, preference–colonization hypothesis, preference–performance hypothesis

## Abstract

The preference–performance hypothesis (PPH) predicts that female insects maximize their fitness by ovipositing on hosts where their offspring perform the best. The preference–performance relationships in bark beetles are complex because before offspring development can occur in the phloem, adult bark beetles must first successfully invade host trees, and then construct galleries beneath the bark. Therefore, a positive correlation between host preference and successful colonization is necessary for the PPH in bark beetles to hold (i.e., the preference–colonization hypothesis in bark beetles). In this study, through field choice experiments, I investigated the successful colonization of the bark beetle, *Polygraphus proximus*, within four allopatrically distributed *Abies* species across a distinct biogeographic boundary in Japan. The results of this study showed that the biogeographic boundary did not limit the successful colonization by *P*. *proximus*. I observed that successful colonization was low in *A*. *firma*, despite it being an exotic species in the study sites and the most preferred at the study sites, indicating a mismatch between preference and colonization success. Additionally, I observed that *A*. *sachalinensis* had a high colonization success rate, even though it was the least preferred species at the study sites.

## Graphical abstract

I investigated the successful colonization of the bark beetle, *Polygraphus proximus*, within four allopatrically distributed *Abies* species across a distinct biogeographic boundary in Japan. The results of this study showed that the biogeographic boundary did not limit the successful colonization by *P. proximus*. High host preference and low colonization success in the exotic host *A. firma* (Host preference–colonization success mismatch I), and low host preference and high colonization success in the other host *A. sachalinensis* (Host preference–colonization success mismatch II) were observed.

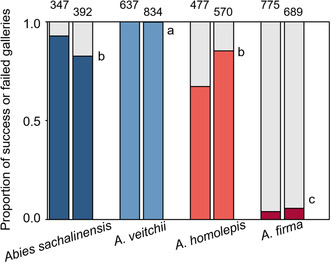

## 1 INTRODUCTION

The preference–performance hypothesis (PPH) predicts that female insects maximize their fitness by ovipositing on hosts where their offspring perform the best (Gripenberg et al., 2010; Mayhew, 2001; Thompson, 1988). A strong correlation between oviposition preference and offspring performance has been reported in multiple insect taxa (Gripenberg et al., 2010). In response to natural selection, adult female host preference and larval performance are adjusted to the local set of potential host plants that render the highest fitness in each population.

Preference–performance relationships in bark beetles are complex because before offspring development can occur in the phloem, adult bark beetles must successfully invade host trees, then construct galleries beneath the bark (Eidson et al., 2018). Therefore, a positive correlation between host preference and successful colonization is necessary for the PPH to hold true in bark beetles (i.e., the preference–colonization hypothesis in bark beetles).

*Polygraphus proximus* is a nonaggressive phloeophagous bark beetle that feeds on Far Eastern *Abies* species (Kerchev, 2014a; Koizumi, 1977; Nobuchi, 1966). The bark beetle is native to throughout Japan, northeastern China, Korea, and the southern Russian Far East (Kerchev, 2014a; Nobuchi, 1966), and it infests freshly cut logs and trees that have been weakened by fire, pathogens, typhoons, or defoliation during the endemic phase of their native range (Koizumi, 1977; Nobuchi, 1966). *P. proximus* has invaded European Russia and West Siberia for the past 10 years, causing mortality across large areas of fir forests (Baranchikov et al., 2010; Kerchev, 2014b). *P. proximus* also caused mass attacks and mortality events in *Abies* trees in its native range in Japan (Chiba et al., 2020; Takagi et al., 2018, 2021; Tokuda et al., 2008). They exhibit a bivoltine cycle in their native distribution (EFSA, 2020; EPPO, 2014). The emergence period and subsequent flight of the first generation of adults occur at the end of spring or at the beginning of summer (May–July), and the second generation emerges from the infested trees at the end of summer (August–September; EFSA, 2020; EPPO, 2014). The mating system of *P*. *proximus* is monogynous (Kerchev, 2014a; Köbayashi & Takagi, 2020). Although a male sex pheromone is suspected to play a role in mating, this role has not been confirmed yet (Kabe, 1959; Nobuchi, 1980).

**FIGURE 1:**
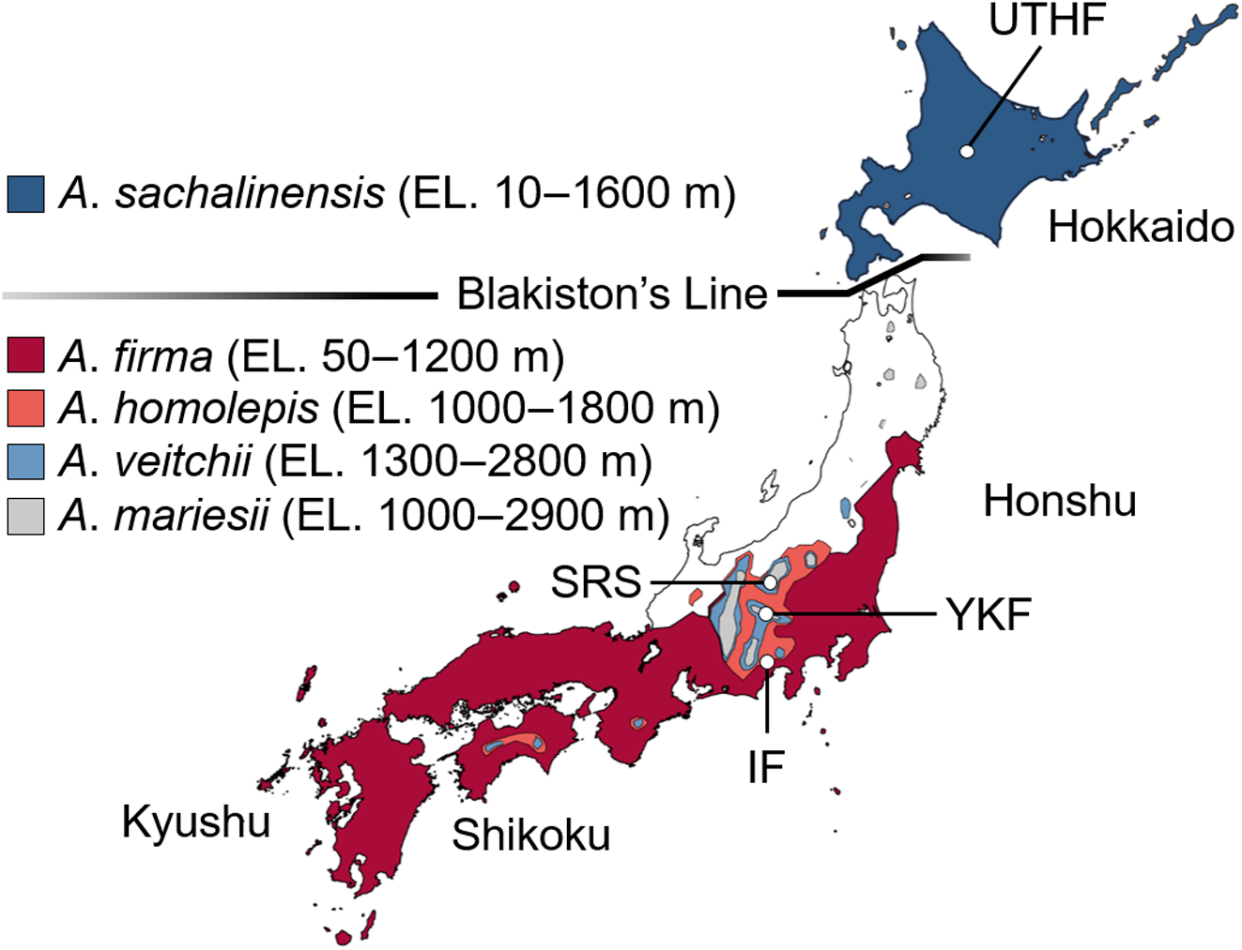
Natural distribution of five *Abies* species in Japan and the locations of the study sites. The natural distributions are based on Tsumura and Suyama (1998). Two tree species can be sympatrically distributed near their boundary areas.

Five native *Abies* species are potential hosts of *P*. *proximus* in Japan (Figure 1). The five *Abies* species in Japan are divided into three phylogenetic groups based on their genetic relatedness: (i) section *Balsamea*, which includes *A*. *veitchii* and *A*. *sachalinensis*, (ii) section *Momi*, which includes *A*. *firma* and *A*. *homolepis*, and (iii) section *Amabilis*, which includes *A*. *mariesii* (Suyama et al., 2000; Tsumura & Suyama, 1998). The five *Abies* species grow in allopatry, with their natural distribution limited in Japan by a distinct biogeographic boundary known as Blakiston's Line; *A. sachalinensis* is the only species native to Hokkaido, the northernmost island in Japan, and not to Honshu, the largest main island in Japan (Figure 1). In contrast, the other four *Abies* species are native to Honshu and not Hokkaido. Thus, the Blakiston's Line has been recognized as a distinct north–south boundary between Hokkaido and Honshu, and the Tsugaru Strait acts as the geographic representation of Blakiston's Line and represents a physical barrier to the movement of many terrestrial species (Aizawa et al., 2007; Haba et al., 2008; Kubota et al., 2014; Suzuki et al., 2004; Tsuyama et al., 2014; Yokoyama & Goto, 2002).

A previous study determined the *P*. *proximus* host preference among four allopatrically distributed *Abies* species in Japan (i.e., *A*. *firma*, *A*. *homolepis*, *A*. *sachalinensis*, and *A*. *veitchii*), and demonstrated that the preference did not vary between Hokkaido and Honshu; *P*. *proximus* preferred *A*. *veitchii* and *A*. *firma* as its hosts, followed by *A*. *homolepis*, with *A*. *sachalinensis* then receiving the lowest preference on either side of the Tsugaru Strait (Takagi, 2022). However, the colonization success of *P*. *proximus* and the differences across biogeographic boundaries which separate host species have not been investigated.

Based on the host preference pattern of *P*. *proximus* and the positive correlation between host preference and successful colonization, I hypothesized that the rate of successful colonization among the *Abies* species would be similar to the host preference, and the geographic barrier separating the *Abies* species would not limit the successful colonization by *P*. *proximus*.

Therefore, this study aimed to determine the proportion of successful colonization of *P*. *proximus* across a biogeographic boundary in Japan, and whether a positive relationship was present between host preference, as shown in the previous study (Takagi, 2022), and colonization success.

## 2 MATERIALS AND METHODS

### 2.1 Study sites

To investigate the successful colonization of *P*. *proximus* on either side of the Tsugaru Strait, two research sites were chosen: one located in central Honshu and the other in Hokkaido (Figure 1). The Honshu site is located at the Sugadaira Research Station (SRS) of the Mountain Science Center, University of Tsukuba, 1300 m above sea level (a.s.l.) in the Sugadaira Highland in Ueda City, Nagano Prefecture, central Japan. The common *Abies* species near the SRS site is *A*. *veitchii*. In the SRS, *A*. *veitchii* and *A*. *homolepis* were introduced from central Honshu, and *A*. *sachalinensis* originated in Hokkaido in the 1960s. The Hokkaido site is located at the University of Tokyo Hokkaido Forest (UTHF) at 300 m a.s.l. in Furano City, Hokkaido, Japan. *A. sachalinensis* is the only *Abies* species native to the UTHF site. In the UTHF, *A. veitchii* was introduced from central Honshu and *A*. *sachalinensis* from Hokkaido in the 1960s. The annual rainfall and average annual temperature at both sites were similar (SRS: 1220.5 mm and 6.6°C; UTHF: 1032.1 mm and 6.7°C, respectively). Outbreaks of *P*. *proximus* on *A*. *veitchii* were reported at both the SRS and UTHF sites (Takagi et al., 2018, 2021), suggesting that the population densities of *P*. *proximus* were high at both sites.

### 2.2 Field experiment

**FIGURE 2:**
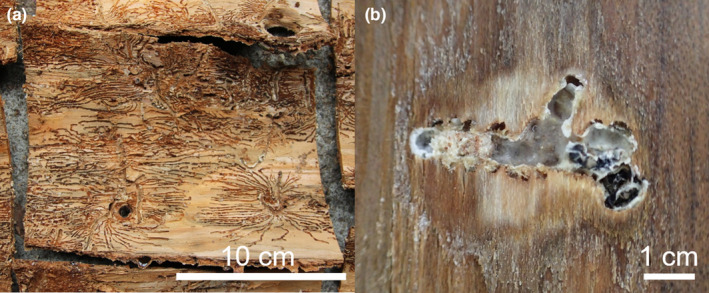
Successful galleries of *Polygraphus proximus* in *Abies veitchii* barks (a) and an unsuccessful gallery in *Abies firma* bark (b).

To investigate the differences in *P*. *proximus* colonization success among the four *Abies* species across the geographic boundary, test logs were collected from *A*. *homolepis* at the SRS, *A*. *sachalinensis* at the UTHF, and *A*. *veitchii* at the Yatsugatake‐Kawakami Forest (YKF), Mountain Science Center, University of Tsukuba, at 1300 m a.s.l. in Nobeyama Highland, Minami‐Saku‐gun, Nagano, central Japan, and *A*. *firma* at Ikawa Forest (IF), Mountain Science Center, University of Tsukuba, 900 m a.s.l. in Shizuoka City, Shizuoka Prefecture, central Japan (Figure 1). Five to six un‐infested trees of each of the four *Abies* species were cut in April 2015 (Table 1). Each tree was cut into 10 logs (i.e., 50–60 logs for each species) with a mean length of 1 m. When obtaining the logs, the associations between the logs and some trees were lost, making it difficult to determine which logs came from which trees. To address this issue, I conducted randomization for the logs in order to minimize the probability of selecting logs from the same tree. At the SRS and UTHF sites, five logs of each species (diameter 8–20 cm) randomly selected were placed vertically on the ground before the swarming period of first‐generation beetles (Table 1). From late June 2015 (after the offspring larvae started to make galleries) to early July 2015 (before the offspring adults emerged from the logs), the bark was peeled from the logs. The number of successful and failed galleries was recorded. Galleries with living offspring were identified as successful galleries (Figure 2a). In contrast, galleries without oviposition, or with oviposition but larval galleries and adults buried in resin were identified as failed galleries (Figure 2b).

**TABLE 1:** Study sites and collection and installation dates of *Abies* species logs used in this study.

Study site	Species	Collection date	Installation date		Latitude/longitude
			UTHF	SRS	
The University of Tokyo Hokkaido Forest, The University of Tokyo, in Furano City, Hokkaido, Japan (UTHF)	*A*. *sachalinensis*	April 20, 2015	April 20, 2015	April 24, 2015	43°10′N/142°23′E
Sugadaira Research Station, Mountain Science Center, University of Tsukuba, in Ueda City, Nagano Prefecture, Japan (SRS)	*A*. *homolepis*	April 21, 2015	April 24, 2015	April 24, 2015	36°31′N/138°21′E
Yatsugatake Forest Station, Mountain Science Center, University of Tsukuba, in Minamisaku County, Nagano Prefecture, Japan (YKF)	*A*. *veitchii*	April 24, 2015	April 27, 2015	April 27, 2015	35°56′N/138°28′E
Ikawa Forest Station, Mountain Science Center, University of Tsukuba, in Shizuoka City, Shizuoka Prefecture, Japan (IF)	*A*. *firma*	April 30, 2015	May 7, 2015	May 2, 2015	35°20′N/138°13′E

### 2.3 Statistical analyses

A generalized linear model (GLM) with a quasi‐binomial distribution and logit link was used to determine the effects of host tree species, study sites, and their interactions on the proportion of successfully constructed beetle galleries. *p*‐values were calculated using *F*‐test. I conducted Tukey's honestly significant difference (HSD) post‐hoc tests to determine differences among the four species and to determine significant effects by tree species. Statistical significance was set at *p* < .05.

All statistical analyses were performed using R 4.0.5 software (R Core Team, 2016), and the “multcomp” (Bretz et al., 2010) and “car” (Fox & Weisberg, 2018) packages.

## 3 RESULTS

**FIGURE 3:**
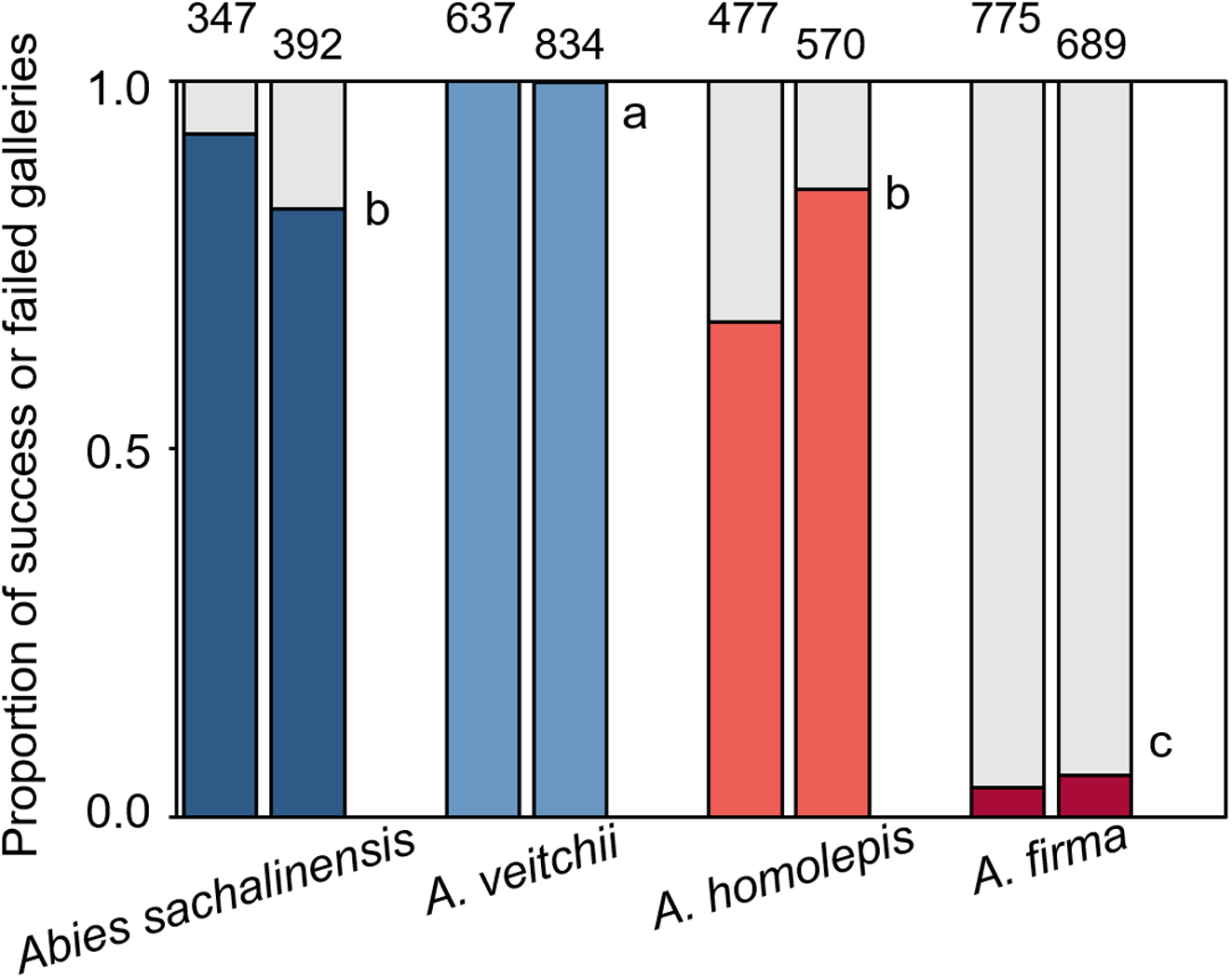
The proportion of successfully (colored) and unsuccessfully (light gray) colonized galleries of *Polygraphus proximus* in four *Abies* species (*A*. *sachalinensis*, *A*. *veitchii*, *A*. *homolepis*, and *A*. *firma*) at the UTHF (left column of each species: The University of Tokyo Hokkaido Forest), and SRS (right column of each species: Sugadaira Research Station) study sites. Means with the same letter are not significantly different, *p* < .05, Tukey Honestly Significant Difference tests. Numerals above each column are the total number of entry holes on each tree species at each study site according to Takagi (2022).

All *Abies* species were attacked by *P*. *proximus* adults at both study sites, and successfully colonized galleries were observed in all *Abies* species on both sides of the Tsugaru Strait (Figure 3). The proportion of successfully colonized galleries differed significantly among the four *Abies* species (*F*_3, 32_ = 143.8, *p* < .001; Figure 3). The proportion of successfully colonized galleries was the lowest in *A*. *firma* (Figures 2 and 3) and the highest in *A*. *veitchii* (Figure 3). The proportion of successfully colonized galleries in *A*. *sachalinensis* and *A*. *homolepis* logs was significantly higher than that in *A*. *firma* logs but lower than that in *A*. *veitchii* logs (Figure 3). No significant difference in the proportion of successfully colonized galleries between the study sites (*F*_1, 32_ = 1.50, *p* = .23), or no significant interaction between sites and species occurred (*F*_3, 32_ = 2.10, *p* = .12).

## 4 DISCUSSION

This study demonstrated the colonization success of *P*. *proximus* within four *Abies* species across a distinct biogeographic boundary separating the *Abies* species in Japan (i.e., the Tsugaru Strait). In the unsuccessful galleries, *P*. *proximus* was buried in the resin, although the defense mechanisms are still unclear from this study. Resin characteristics, such as resin flow, chemistry, viscosity, and crystallization, may affect colonization behavior and success. The results showed that the order of colonization success among the *Abies* species was not significantly different on either side of the Tsugaru Strait. These findings are consistent with those of Takagi (2022) reporting that the Tsugaru Strait does not represent a geographic boundary of host preferences for *P*. *proximus*, indicating that host specialization has not occurred across the Tsugaru Strait.

According to the PPH and a positive correlation between host preference and successful colonization (i.e., the preference–colonization hypothesis) in bark beetles, the rates of successful colonization among the *Abies* species would be reflected by their host preference. Specifically, *A*. *veitchii* and *A*. *firma* were expected to exhibit the highest successful colonization rates, followed by *A*. *homolepis* and *A. sachalinensis* exhibiting the lowest rate. However, this study showed that the successful colonization rate was the highest in *A*. *veitchii*, followed by *A*. *sachalinensis* and *A*. *homolepis*, and was the lowest in *A*. *firma*, indicating a preference–colonization mismatch.

*Polygraphus proximus* prefers *A*. *firma* logs on both sides of the Tsugaru Strait (Takagi, 2022). However, in the present study, the successful colonization rate of *P. proximus* in *A*. *firma* logs was the lowest, and *P*. *proximus* was killed by the resin in *A*. *firma* logs immediately after entry. In contrast, a previous study conducted in Kyushu, the main southwest island in Japan, showed that *P. proximus* successfully colonized the bark of trees defoliated by the curculionids *Parendaeus abietinus* Kojima and Morimoto, resulting in the mass mortality of *A. firma* (Tokuda et al., 2008). Based on the distribution of the *Abies* species (Figure 1), neither of the *P*. *proximus* populations observed in the present study had previously encountered *A*. *firma*. Thus, *P. proximus* populations in the present study likely had little evolutionary experience with *A*. *firma* and a poor estimation of its suitability, resulting in a preference–colonization mismatch (Jaenike, 1978).

This study also showed that the successful colonization rate in *A*. *sachalinensis* was high (71.2%–100%). *A. sachalinensis* is the only native *Abies* species at the UTHF site. Nevertheless, it was the least preferred host among the four *Abies* species (Takagi, 2022). This weak preference–colonization relationship in *A*. *sachalinensis* appears to be another mismatch between host preference and colonization success. The larval performance in *A*. *sachalinensis* may shed light on this mismatch.

Taxonomic proximity among host tree species may influence host preference and the larval performance of bark beetles (Bertheau, Salle, Rossi, et al., 2009; Bertheau, Salle, Roux‐Morabito, et al., 2009). As a result of feeding on plants that are phylogenetically distant from their preferred hosts, insects generally lose fitness (Bertheau et al., 2010). However, no positive correlation between host taxonomic proximity and host preference in *P*. *proximus* was observed in a previous study (Takagi, 2022). In contrast, this study showed that the relationship between taxonomic proximity and colonization success is complex. The successful colonization rate in *A*. *sachalinensis*, which belongs to the section *Balsamea*, was not significantly different from that in *A*. *homolepis*, which belongs to the section *Momi*. Conversely, the successful colonization rate of *A*. *veitchii*, which belongs to the *Balsamea* section, was the highest among the four *Abies* species, whereas that of *A*. *firma*, which belongs to the *Momi* section, was the lowest. These results suggest that there is no positive correlation between host taxonomic proximity and colonization success in *P*. *proximus*.

Most preference–performance studies have been carried out on lepidopteran and dipteran species, resulting in skewed information that conforms to the theory (Gripenberg et al., 2010). A previous study reported a lack of correlation between preference and performance in monophagous insects compared with that in oligophagous and polyphagous species, probably because of the small number of studies on this group of insects (Gripenberg et al., 2010). A few studies have reported positive preference–performance relationships in monophagous bark beetles (Bertheau, Salle, Rossi, et al., 2009; Bertheau, Salle, Roux‐Morabito, et al., 2009; Eidson et al., 2018). Other studies have shown a negative preference–performance relationship in the bark beetle *Dendroctonus ponderosae* Hopkins, commonly known as the mountain pine beetle (MPB) (Bentz et al., 2015; Raffa et al., 2013). The MPB was more likely to attack its historical host, the lodgepole pine, *Pinus contorta* Douglas, than its naïve host, whitebark pine, *Pinus albicaulis* Engelm., despite the inferior defensive capabilities of the whitebark pine. In contrast, in the present study, *P*. *proximus* from the UTHF site did not preferentially attack *A*. *sachalinensis*, which is the historical and sole native host in Hokkaido (i.e., the UTHF sites), although the successful colonization rate in *A*. *sachalinensis* was high. The present study also showed that *P*. *proximus* preferred the naïve host *A*. *firma*, despite having the lowest successful colonization rate in *A*. *firma*. Thus, in the present study, I demonstrated a different pattern of preference–colonization mismatches in bark beetles, although the mechanisms underlying the preference–colonization mismatch and preference–performance relationships remain unclear.

While this study demonstrated the mismatches between host preference and colonization success in bark beetles, it is worth noting that freshly cut logs were used in this study. Although *P*. *proximus* in the unsuccessful galleries were found buried in resin, logs, while containing some residual resin, are largely undefended substrates, incapable for example of induced defenses. The volatilization rates of defense compounds may differ between logs and standing trees. Further studies are necessary to determine the preferences and colonization success using standing trees.

## AUTHOR CONTRIBUTIONS

**Etsuro Takagi:** Conceptualization (lead); data curation (lead); formal analysis (lead); funding acquisition (lead); investigation (lead); methodology (lead); project administration (lead); resources (lead); visualization (lead); writing – original draft (lead); writing – review and editing (lead).

## FUNDING INFORMATION

This work was supported by JSPS KAKENHI, Grant number 19K15874.

## CONFLICT OF INTEREST STATEMENT

The author declares no conflicts of interest.

## Data Availability

All files related to this study are available from the DRYAD at https://doi.org/10.5061/dryad.vq83bk3wh (Takagi, 2023).
